# Changes of the Oral Secretions

**Published:** 1890-03

**Authors:** G. T. Thurer

**Affiliations:** U. of M.


### Changes of the Oral Secretions.

BY G. T. THURER, U. OF M.

A thorough knowledge of the fluids of the mouth is indispensable to the dentist if his aspirations look higher than the mere insertion of artificial dentures, or the filling of teeth, as they of necessity mingle with and lubricate our food, and exert an appreciable influence over digestion, and all of them may, in disease, materially interfere with this physiological function.

Moreover, none of the secretions of the alimentary tract have a higher claim upon the attention of the dentist, looked at from a physiological or pathological stand-point, than the saliva, as it is claimed to be the fluid, which, when abnormal, assists, if it does not produce, caries of the teeth. The properties and characteristics of the oral secretions, though not included within the limits of this subject, will be briefly considered.

The saliva is composed of the secretions of the parotid, submaxillary and sublingual glands, also of the mucous follicles.

The parotid saliva is a clear liquid, not viscous, and slightly alkaline. It gives no reaction for mucin, but contains albumen, ptyalin, and among the more or less peculiar constituents we find paraglobulin, caporic acid, urea, and traces of sulphates. The reaction of the first secreted parotid saliva is less alkaline than that secreted later. On standing, the parotid secretion becomes turbid, owing to the escape of carbonic acid and the consequent precipitation of calcium carbonates.

The submaxillary saliva differs from the parotid saliva in being more alkaline, and from the presence of mucus, more viscid; it contains, often in abundance, salivary corpuscles and amorphous masses of proteid material. The character of submaxillary saliva depends upon the exciting stimulus to its secretions; stimulation of the chordatympain nerve causes a normal, rich, alkaline secretion, as noticed when acids are applied to the surface of the tongue, but in it no ptyalin is found; with long-continued stimulation the organic solids diminish somewhat, though at first the mucin is slightly increased. Stimulation of the sympathetic nerve supplying the gland, or an application of pepper or alkalies to the tongue, produces a strongly alkaline secretion of high specific gravity (1.007 to 1.018) but viscid, turbid, slowly-flowing, rich in mucus, and irregularly formed cell elements. In paralysis of the nerves supplying the gland very watery saliva is found containing little solids or mucus. This variety is known as paralytic saliva. The submaxillary saliva is more alkaline and viscid than that of the parotid and its average specific gravity is from 1.002 to 1.003. It contains much more carbonic acid than venous blood but is poorer in nitrogen. The sublingual saliva is very viscous and thready, strongly alkaline, rich in mucus and salivary corpuscles, and would appear to be the richest in solids of all salivas. Traces of cholesterin and fat have been found in it. The secretion of the mucous follicles is a mucus which is viscid in character, grayish in color, and contains, when free from admixtures with other secretions, epithelial cells and leucocytes. Its reaction is, according to Claude Bernard, alkaline, but when normal becomes markedly acid. The physical characteristics of mixed saliva are that it is tasteless, colorless, odorless, specific gravity 1.002 to 1.006, reaction is alkaline, appearance is generally turbid, consistence glassy and frothy. On standing for some hours in a cylindrical glass vessel an opaque, whitish deposit collects at the bottom, while the supernatant fluid becomes clear and of a faint bluish tint.

The secretion of saliva is not constant, even normally. Its secretion may be excited by the sight or even thought of food, especially when hunger exists; by the movements of mastication —the secretion of the parotids alternating the parotid of the side on which mastication is going on, secretes at least one third more than that of the other side, and when mastication is changed to the other side, the corresponding parotid goes vigorously to work, while the action of the first partially ceases. Secretion is also increased by vapors of ether or acetic acid, or by electrical stimulation. Nausea will generally excite a free flow of the buccal fluids; at such times the mucous follicles or glands become especially active.

The various mental conditions operate variously upon the salivary glands; fear diminishes the secretion very much; fright may check it entirely; anger also diminishes it, while good humor and mirth increase the secretion. Erotic emotions increase the secretion of the mucus considerably, and that of the saliva to some extent. Grief augments the secretion of saliva and mucus. Fluctuations in the quantity of water in the blood has but little influence upon the amount of saliva elaborated in a given period. Neither does it change the relative proportions of the solid constituents of the saliva. Dry food also acts as a stimulus, and increases the amount of secretion to a much greater extent than food containing moisture. There is also an increase in quantity when patient is suffering from debility, confluent small-pox, ague, hysteria, facial neuralgia, and organic diseases of the stomach or abdominal viscera. The action of certain drugs will also produce this effect, and chief among these we have mercury, pilocarpin, eserin, iodide of potassium, etc. During dentition, the first indication of the advance of the teeth is an increased flow of saliva—a healthy manifestation, as it serves to keep the mouth moist and cool. The increased flow of saliva, called "drooling," is doubtless due to irritation of the tri-facial nerve, which is sensory to the teeth and nutrient to the salivary glands. Pregnancy, a purely physiological function, giving rise to repeated vomiting and capricious appetite, disturbs the buccal fluids to such a degree that a rapid decay of the teeth is observed as a consequence.

The saliva is subject to many and great changes by disease. The saliva, proper, may be changed from its natural to a definitely acid secretion, or it may be changed to more than its alkaline condition. This latter is exhibited when salivary calculus is rapidly deposited upon the teeth. Any change of the mucus is usually to an increased acidity. When this condition is produced to such an extent that it is not neutralized by the saliva, as is generally the case when green stain is deposited upon the teeth, it becomes a source of mischief.

The saliva becomes changed to an abnormal or diseased condition in two ways; first, by a vitiated state of the blood, and second, by a diseased condition of the glands themselves; the latter is often complicated with the former. The saliva, proper, is more frequently affected by a deteriorated state of the blood, but the mucus by an unhealthy or diseased condition of the mucous follicles. These latter being situated in the mucous membrane, near the surface, are more exposed than the salivary glands to the influence of local irritating causes. The salivary glands are often required to perform more than their natural amount of labor, sometimes from local causes or stimuli, as in the use of tobacco or other similar agents; from constant use of jaws in mastication, and from general constitutional causes, as in ptyalism and in various vitiated conditions of the blood. When any organ is overtaxed its production is necessarily deteriorated. It is a well-known fact that when the saliva is neutral or slightly alkaline caries of the teeth is at a stand-still, and when the reverse is the case decay is progressing more or less rapidly, depending upon the character of the secretion. Simple calciferous saliva, of itself, does not particularly harm the teeth, but the latter complications are not to be so characterized; in addition to covering a portion of the teeth with calculus it may give rise to ranula and catarrhal inflammation of the gums, which latter dissease, if allowed to care for itself, permits tartar to be deposited, even on the apex of the root of the tooth, causing neuralgia, but before the latter complication the whole secretion is changed by becoming profuse in quantity and excessively alkaline in its reaction, denoting disturbance of the nervous equilibrium. Saliva presents the same reaction during putrid fever and purpura hemorrhagica. Pending the formation of an alveolar abscess up to the climax of pain, the saliva is abundant and the reaction alkaline or neutral, but afterward it becomes acid until the subsidence of the swelling, when it once more becomes normal in quantity and quality. While in general the saliva may give an acid reaction as the result of inflammation of the gums, we do not look to it, but to the hypersecretion of mucus as the cause of such reaction. The acute diseases causing hypersecretion of mucus are acute stomatitis, bronchitis, pneumonia, pharyngitis, pleurisy, and acute enteritis, while those of a chronic nature are marsh-fever, dyspepsia, gastralia, dysentery, etc. The mucus secreted is not of itself to be feared in these affections, but by increasing the flow of saliva by the use of remedial agents, there is a large amount of ptyalin present, which, in conjunction with the mucus, furnishes an active ferment, producing from azotized substances, as secondary reactions, butyric, benzoic, valeric and other acids destructive to the teeth. In simple gastric disorders, saliva is changed to an acid condition, which may be accounted for by increased tissue metamorphosis, the epithelium of glandules being cast off so rapidly that a chemical change takes place in the cells causing the secretion to appear acid.

In febrile diseases the secretion of the saliva is very much diminished, and sometimes checked altogether; then the secretion of mucus being kept up, the teeth suffer injury, for the mucus at such times is always in a vitiated condition and changes rapidly to a worse condition while in the mouth. The water evaporates and leaves a thick, tenacious sordes covering the teeth and lining the mucous membrane of the mouth. This condition also facilitates the decomposition of foreign material that may be brought in contact with it.

Saliva is very copious, frothy and alkaline in apoplexy, hysteria and hydrophobia. We can find parasites in the saliva constantly—as the thrush fungus in infancy, and in the wasting diseases, as tuberculosis, also micrococcus dentalis in wooping-cough, and leptothrix-buccalis, vibrios, and bacteria are found at almost any period. The latter stimulate the nitrogenous remains of food to putrefactive fermentation, yielding lactic acid from sugar, etc. This acid is present in saliva in gout, rheumatism, intermittent fever, and diabetes; acetic acid in scrofula, small-pox, scorbutus, and aphthæ; hydrochloric acid from the immoderate use of animal flesh, including salt meats, galvanic action, and in the earlier stages of inflammation of the oral mucous membrane there is an increase of the soluble chlorides, which, when under favorable conditions, decompose, liberating the chlorine, which unites with the hydrogen present to form the above acid.

Saliva is changed to fetid condition in cancrum oris and syphilitic ulcerations, and is usually acid in reaction in catarrhal inflammation, cutaneous diseases, and venereal affections; the gums in the latter are always softened and spongy unless mercury has been exhibited. In mercurial salivation and iodism as much as two per cent. of inorganic matter with a large quantity of coagulable material have been secreted, the latter decomposing rapidly when lodged between the teeth, and presenting an acid reaction.

In diabetes there is atrophy of the glandular organs of the mouth, throat and gums; saliva strongly acid, and the mucous membrane most likely to be the seat of scorbutus. Ammonia and sulphuretted hydrogen are nearly always present in the saliva of unclean mouths.

It is not to be wondered at that caries of the teeth should be so prevalent when it is remembered that no disturbance of the human system prevails, for even a short period, without changing the character of the oral secretions. The various acids, organic and inorganic, coming in contact with the teeth as ailments or remedial agents, or as the result of fermentation, which latter is favored in proportion as the saliva is scanty and the mucus abundant, renders it necessary for the dentist of to-day and his future successor to be a student of chemistry and hygiene—a preserver and master of the laws of health.

